# Physical activity across days of week, video games, and laptop use are more likely to influence weight gain among Saudi Youth

**DOI:** 10.3389/fspor.2022.963144

**Published:** 2022-08-30

**Authors:** Mohammed Shaab Alibrahim

**Affiliations:** Department of Physical Education, College of Education, King Faisal University, Al-Ahsa, Saudi Arabia

**Keywords:** adolescents, body mass index (BMI), physical activity, in-school, out of school, sedentary behaviors, screen time, children

## Abstract

**Purpose:**

The purpose of this study was to determine the extent and prevalence of daily PA and the sedentary behavior during inactive spare-time among young Saudis and their effect on overweight and obesity.

**Methods:**

A total of 357 students completed all stages of this study. They included 282 boys aged 11.95 ± 0.73 years (213 children aged 11.62 ± 0.506 years and 69 adolescents aged 13.16 ± 0.373 years) and 75 girls (16 children aged 11.94 ± 0.236 years and 59 adolescents aged 13.47 ± 0.626 years). For each participant, height and weight were measured, and BMI was calculated. A questionnaire asking about daily PA and sedentary habits during the previous 7 days was also used. Multiple comparisons were performed to test for differences between groups by sex and age category, and stepwise multiple regression analysis was used to determine which variables significantly affected BMI.

**Results:**

The overall prevalence of overweight and obesity was 17.02 and 28.37% in boys and 9.33 and 0% in girls, respectively. The patterns of PA were changing within elements and between sexes. Both groups of boys were more active than girls during breakfast and lunch periods (*p* < 0.001 for all). A significant difference in favor of boys was also observed between the two groups of boys and the group of adolescent girls regarding after-school physical activity (*p* < 0.05 for all). Prepubescent boys reported being generally less active during the previous 7 days than their female counterparts and adolescent peers. Sedentary behaviors did not differ between all groups. Playing video games and using laptops were the parameters significantly associated with BMI.

**Conclusions:**

This study noted that the prevalence of overweight and obesity was more pronounced in boys than in girls. However, boys were more active but had the same degree of sedentary behavior as girls. Physical activity across the days of weekly, video games, and laptop use were the parameters that most affected BMI.

## Introduction

Over the past decades, obesity has become one of the main factors contributing to the global burden of non-communicable diseases. Relevant studies have noted significant association of obesity with type 2 diabetes, hypercholesterolemia, hypertension, lung disease, rheumatoid arthritis, sleep apnea, colon disease, and thyroid disease (Althumiri et al., 2021). Simmonds et al. (2016) also suggested that around 55% of childhood obesity cases result in adolescent obesity, of which 80% occurs in adulthood. The world health organization estimated that obesity will affect up to 20% of the world's population by 2050 (Davis et al., 2022). Therefore, preventing and controlling childhood obesity is important to promote good health and reduce high risk of chronic diseases in adulthood (Simmonds et al., 2016). In this sense, several indices are commonly used to assess obesity, including the body mass index (BMI), which is the most widely used reliable tool given the limitations of other indices used to measure obesity in clinical practice (Flynn et al., 2022). However, in children and adolescents, this index has some errors related to the rapid and variable physical growth that depends on age, sex and race. To address the observed disparities, health organizations have adopted gender- and age-specific body weight guidelines and used them to diagnose obesity. It has also been suggested that low physical activity (PA) levels are a leading cause of childhood and adolescent obesity (Flynn et al., 2022) and the fourth risk factor for global mortality (World Health Organization, 2009).

Benefits of regular physical activity (PA) are numerous and include preventing excess weight gain, improving the functions of the heart and lungs, combating health conditions and diseases, improving mood, and increasing muscle strength and endurance (World Health Organization, 2017; U.S. Department of Health Human Services, 2018). According to the World Health Organization 2020 guidelines on PA and sedentary behavior (Bull et al., 2020), all adults should undertake 150–300 min of moderate-intensity PA, 75–150 min of vigorous-intensity PA, or some equivalent combination of moderate-intensity and vigorous-intensity aerobic PA per week. Children and adolescents (in the 5–17 age range) should get at least 60 min of physical activity per day, including moderate-to-vigorous aerobic activity and age-appropriate muscle and bone strengthening. Furthermore, the total amount of physical activity appears to be more important than any other component of exposure (frequency, duration, etc.) or combination of activities (aerobics, strengthening) in terms of health benefits for adolescents. Children and adolescents should also minimize the time spent in extended periods of sedentary activity (Piercy et al., 2018).

Alasqah et al. (2021) noted that most Saudi adolescents do not meet the recommendation of 60 min of PA per day; consequently, 28–30% of adolescents are overweight or obese. Maintaining regular PA is especially important for children and adolescents not only because it provides health benefits, but also because it is an important factor in acquiring lifelong habits (Ridgers et al., 2007) and for a public health priority (Dallman et al., 2009). In fact, adolescents spend up to 25% of their time at school, which provides them with opportunities to engage in PA during the day (Jago and Baranowski, 2004). Consequently, the school is an ideal environment for increasing PA through physical education and recess (Mounesan et al., 2012).

Physical education (PE) class has been shown to be an opportunity for school children to engage in physical activity (McKenzie et al., 2004; Jago et al., 2009). It was also suggested that PE can help increase schoolchildren's daily PA and reduce sedentary time (Mayorga-Vega et al., 2018). PE accounts for 6–23% of daily activity in children and adolescents (Cardon et al., 2004). Aljuhani and Sandercock (2019), in an accelerometry-based study, noted that school children spent 22% of their PE class time in moderate to vigorous PA. These authors further showed that 40 and 24% of schoolchildren achieved recommended levels of physical activity on PE and non-PE days, respectively, and that PE days outperformed non-PE days with 12.9, 14.7, and 14.8 min more for all, inactive, and unfit schoolchildren, respectively. Tudor-Locke et al. (2006) reported that lunchtime contributed to 15–16% of PA, exceeding the contribution of break time (8–9%) and PE classes (8–11%). They concluded that lunchtime PA was potentially the most important source of daily PA. All children have the ability to be active during lunchtime, as they may choose which activities they participate in and might engage in unstructured PA (Biddle et al., 2004).

Electronic devices and video games, characterized by low energy consumption, are also known to affect the PA levels of children and adolescents (Bartosiewicz et al., 2020). Wells et al. (2008) showed that these behaviors often occur in the context of sleep disturbances and lead to increased body fat, increased weight, and vital signs in children and adolescents. Bartosiewicz et al. (2020) expressed that the solution lies in the promotion of daily physical activity, healthy eating, and adequate sleep, which would also promote the goal of improving mood and emotional health. The present study aims to determine the prevalence of physical activity and sedentary behaviors (the use of television, tablets, laptops, game consoles, video games, and smartphones) and their effect on overweight and obesity in a group of youth aged 11–14 years in the Al-Ahsa governorate.

## Materials and methods

### Participants

A multi-stage cluster random sampling method was used to select 300 boys and 300 girls from middle schools in Al-Ahsa governorate located in the Eastern Province. The details of the sample size estimation have been published previously (Said and Shaab Alibrahim, 2022). In all, 450 positive responses were received from parents, among which 93 students were excluded due to sever obesity (*N* = 06), inability to complete all tests (*N* = 04), incorrect responses (*N* = 09), or lack of interest (*N* = 74). Finally, a total of 357 students completed all stages of this study. They included 282 boys (78.99%) aged 11.95 ± 0.73 years, including 213 children (11.62 ± 0.506 years; 75.53%) and 69 adolescents (13.16 ± 0.373 years; 24.47%), and 75 girls (21.01%), including 16 children (11.94 ± 0.236 years; 21.33%) and 59 adolescents (13.47 ± 0.626 years; 78.67%). All participants were randomly selected to participate in this study, and written consent was signed by their parents prior to any measurements being taken. The protocol was approved by the Ethics Committee of the Deanship of Scientific Research, King Faisal University (Ref. No. KFU-REC-2021-OCT-EA00019).

### Protocol

Assessments were conducted during regular physical education classes. They included completing a pre-administered questionnaire and measuring each participant's height and body weight. All assessments were conducted in the schools' sports halls by the PE teachers with an average of 30 participants per period. The questionnaire focused on students' PA and behaviors related to sedentary lifestyles. It consisted of three parts and took about 25 min to complete. The first part covered demographic data (age, nationality, and sex); the second part consisted of an activity questionnaire for older children (PAQ-C; Kowalski et al., 2004); and the third part asked about sedentary behaviors, using the survey on physical activity and school nutrition NSW (Hardy et al., 2011). Full instructions and explanations were given to the students to help them complete the questionnaire properly. Those who had difficulty understanding any concept regarding the data or were confused by the questionnaire choices were assisted by a researcher who attempted to explain unclear concepts or instructions.

### Study outcomes

#### Anthropometry

For each participant, height and weight were measured, and body mass index was calculated. Height was measured barefoot using a Harpenden stadiometer (Holtain, Crymych, Wales) with a degree of accuracy of 0.1 cm. Weight was determined using a pan scale (Seca, Hamburg, Germany) with minimal clothing and to the nearest 0.02 kg. BMI was calculated as weight (kg)/height squared (m^2^). The data were entered into the anthropometric calculator V.3.1 of the World Health Organization (WHO; Geneva) software Anthro plus V.1.0.4 (World Health Organization, 2016) and participants were classified into four categories according to WHO's growth reference data for 5–19 year olds (World Health Organization, 2007): (1) underweight (UW; BMI-for-age <5th percentile), (2) normal weight (NW; 5th percentile ≤ BMI-for-age <85th percentile), (3) overweight (OW; 85th percentile ≤ BMI-for-age <95th percentile), and (4) obese (OB; BMI-for-age ≥95th percentile).

#### Physical activity level

The Physical Activity Questionnaire for Older Children (PAQ-C) is a 7-day, self-administered recall tool designed to provide students in grades 4–8 with general reports on PA levels during the elementary school year (Kowalski et al., 2004). In order for it to be easily understood by all participants, the English version of the PAQ-C was first translated into Arabic by two bilingual translators, and a standardized version was developed. This version was then translated back into English by two English-speaking translators of Arabic origin, and both versions (Arabic and English) were reviewed by five bilingual lifestyle experts. The necessary adjustments requested by the experts were made and the reliability of the Arabic version was verified. Cronbach's PAQ-C alpha value was 0.788, which is close to that of Sirajudeen et al. (2022) and indicates a good level of reliability of the final version in Arabic.

The PAQ-C consisted of ten items, nine of which were used to provide a summary score of PA, called the “*Overall PA score*.” The tenth item assessed whether endogenous or exogenous factors had prevented the child from regularly engaging in PA during the previous week. Each of the first nine items was rated on a five-point scale, with the lowest response being rated 1 and the highest being rated 5. Item 1 was related to leisure time activities; the average of all activities on the checklist was called the “*Spare-time PA Score*.” Items 2 through 9 were related to activities done during PE class, recess, lunch, right after school, evening, weekend, which best describes you, and how often you did physical activity each day in the past week? The average of the responses was calculated and reported as the “*PA across days of week score*” (Kowalski et al., 2004).

The overall PA score obtained was used to classify participants into five categories: (1) very little active (score between 1 and 1.79), (2) little active (score between 1.8 and 2.59), (3) moderately active (score between 2.6 and 3.39), (4) active (score between 3.4 and 4.19), and (5) very active (score between 4.2 and 5; Pimentel, 2010).

#### Sedentary behaviors

Sedentary behaviors were assessed through a single question containing five items regarding the use of electronic devices and the Internet (iPad, tablet, computer, or smartphone); watching television or movies or programs on the Internet; and playing games (on a computer, game console, smartphone, or iPad). Possible responses included: not at all, scored 0; <30 min per day, scored 1; more than 30 min but <1 h per day, scored 2; more than 1 h but <2 h per day, scored 3; more than 2 h but <4 h per day, scored 4; and more than 4 h per day, scored 5. An average score was calculated by summing a participant's scores and dividing by the total number of entries. The average score obtained was used to classify participants as not sedentary, mildly sedentary, sedentary, highly sedentary, or very highly sedentary. The recommended duration of sedentary activity is 2 h or less per day, and performing sedentary activities for more than 2 h per day is considered high sedentary activity.

In the Saudi context, weekdays are Sunday through Thursday, and weekends include Friday and Saturday. The school day is defined as the time between when the school bell rings to begin the school day and when it rings to announce the end of the school day. This time is generally between 7:00 am and 12:30 pm. After-school time is defined as 120 min after the end of the school day, and it is designed to include the ride from school and the period immediately after students arrive at home. Evening refers to the entire period from the end of after-school time until 8:00 p.m, which is the 300 min between 3:00 and 8:00 p.m. each weekday.

### Data analysis

All statistical calculations were performed using SPSS version 26. Descriptive statistics (mean and standard deviation) were used to summarize participant characteristics, and *p*-value was set at 0.05. The normality of distributions was checked using the Shapiro-Wilk test and the homogeneity of variance was assessed using Brown-Forsythe test for the assumption of equal variances. Brown-Forsythe's *p*-values indicated that the homogeneity of variance assumption was violated in all variables except PA score in leisure time and the overall sedentary behavior score. Chi-square tests were used to test differences between categorical variables, while Welch's *t*-test and Welch's ANOVA were used for continuous variables. The Games-Howell *post hoc* test was used to test between-group differences. The linearity of the predictor variables was tested using scatter plots. The normality and homoscedasticity of residuals were also tested. The multicollinearity of independent variables was tested using variance inflation factor (VIF) values, all of which were <5. Stepwise multiple regression analysis was used to determine the association between physical activity and sedentary behavior and BMI of participants stratified by sex and age category. To avoid confounding, two models were run: one with the overall PA score and sedentary behavior score and the other with the spare-time PA score, the PA score across days of the week, and the sedentary activity levels. Results are presented as unstandardized beta coefficients (β and standard error) and R-squared values.

## Results

### Anthropometry

A total of 357 students completed all stages of the study and their data were retained for analysis. They included 75 girls aged 13.15 ± 0.85 years (16 children aged 11.94 ± 0.236 years and 59 adolescents aged 13.47 ± 0.626 years) and 282 boys aged 11.95 ± 0.73 years (213 children aged 11.62 ± 0.506 years and 69 adolescents aged 13.16 ± 0.373 years). Welch's *t*-test showed a significant difference between boys and girls in height (*p* < 0.001), weight (*p* < 0.001), and BMI (*p* < 0.05). Multiple comparisons also showed that prepubertal boys were smaller and lighter than both groups of girls (*p* < 0.05 for prepubescent girls' weight; *p* < 0.001 for the rest). Adolescent boys were also smaller (*p* < 0.001 for both) but as heavy as both groups of girls. Prepubescent girls were less heavy than their adolescent counterparts (*p* < 0.01) and had the lowest BMI compared with the other groups (*p* < 0.001 for prepubertal boys; *p* < 0.01 for others). No significant differences were noted between the boy groups in BMI and between the girl groups in height (Table 1).

**Table 1:** Age, anthropometry, and physical activity and sedentary behaviors scores for Saudi students stratified by sex and age category.

	**Boys**		**Girls**				**Between groups by sex and age**	
							**category sex and age category**	
	**Children (*N* = 213)**	**Adolescents (*N* = 69)**	**Children (*N* = 16)**	**Adolescents (*N* = 59)**	**Total boys (*N* = 282)**	**Total girls (*N* = 75)**	**Welch F**	***P***
Height (m)	1.41 ± 0.09^c^^,^^d^	1.44 ± 0.10^c^^,^^d^	1.57 ± 0.05^a^^,^^b^	1.59 ± 0.07^a^^,^^b^	1.42 ± 0.09	1.59 ± 0.06^***^	115.676	0.001
Weight (kg)	40.35 ± 11.58^c^^,^^d^	45.10 ± 15.79	44.28 ± 3.18^a^^,^^d^	49.03 ± 7.05^a^^,^^c^	41.59 ± 12.94	47.92 ± 6.65^***^	15.001	0.001
BMI (kg.m^−2^)	20.25 ± 5.15^c^	21.38 ± 6.14^c^	17.95 ± 1.01^a^^,^^b^^,^^d^	19.30 ± 2.41^c^	20.54 ± 5.43	18.99 ± 2.24^***^	13.109	0.001
Spare-time PA score	1.87 ± 0.49	1.91 ± 0.52	1.83 ± 0.66	1.74 ± 0.46	1.88 ± 0.49	1.76 ± 0.51^*^	2.638	NS
PA across days of week score	2.86 ± 1.10^d^	2.90 ± 1.19^d^	2.18 ± 1.08	2.19 ± 0.83^a^^,^^b^	2.87 ± 1.12	2.19 ± 0.88^***^	11.329	0.001
Overall PA score	2.61 ± 0.67^c^^,^^d^	2.54 ± 0.68^c^	2.16 ± 0.60^a^^,^^b^	2.33 ± 0.54^a^	2.59 ± 0.67	2.29 ± 0.56^***^	6.464	0.001
Sedentary behaviors Score	2.69 ± 0.92	2.97 ± 1.03	3.07 ± 1.19	2.89 ± 0.95	2.77 ± 0.96	2.93 ± 1.00 NS	1.639	NS

Data are means ± standard deviation.

a Differs to children boys using Welch's ANOVA analysis;

b Differs to adolescent boys using Welch's ANOVA analysis;

c Differs to children girls using Welch's ANOVA analysis;

d Differs to adolescent girls using Welch's ANOVA analysis.

* *p* < 0.05,

*** <0.001 differs to boys using Welch's *t*-test. BMI, body mass index; PA, physical activity; NS, not significant.

Referring to the sex-specific BMI-for-age percentile charts, the prevalences of overweight and obesity were 17.02% (19.25% of children and 10.14% of adolescents) and 28.37% (26.76% of children and 33.33% of adolescents) for boys; for girls, the prevalences of overweight and obesity were 9.33% (0% of children and 11.86% of adolescents) and 0%, with significant differences between BMI categories (*X*^2^ = 56.966; *p* < 0.001; Table 2).

**Table 2:** Prevalence of overweight and obesity, physical activity, and sedentary behaviors among Saudi students stratified by sex and age.

		**Boys**		**Girls**		**Chi square**	
		**Children**	**Adolescents**	**Children**	**Adolescents**	**χ^2^**	***P***
Body mass index	Underweight	6.57	20.29	0	13.56	56.966	0.001
	Normal weight	47.89	33.33	100	76.27		
	Overweight	19.25	10.14	0	11.86		
	Obese	26.76	33.33	0	0		
Physical activity	Slightly active	18.31	21.74	43.75	27.12	34.344	0.001
	Moderately active	56.81	53.62	50	61.02		
	Active	21.13	23.19	6.25	11.86		
	Highly active	3.76	1.45	0	0		
Sedentary behaviors	Not sedentary	8.92	15.94	18.75	10.17	27.18	0.007
	Mildly sedentary	19.72	26.09	31.25	32.20		
	Sedentary	46.48	36.23	18.75	35.59		
	Highly sedentary	20.66	18.84	25.00	22.03		
	Very highly sedentary	4.23	2.90	6.25	0.00		

Results are expressed in percentage.

### Overall physical activity score

A significant difference in favor of boys was noted between the boys' and girls' groups regarding overall PA score (*p* < 0.001). In addition, Welch's ANOVA revealed a significant superiority of the prepubescent boys' groups over the two girls' groups (*p* < 0.05 for prepubescent girls; *p* < 0.01 for adolescent girls). Significant difference was also noted between adolescent boys and prepubescent girls (*p* = 0.05). No significant differences were noted between same-sex groups (Table 1). PA prevalence differed significantly between groups. Most boys were moderately active (56.81% for children and 53.62% for adolescents) or active (21.13% for children and 23.19% for adolescents). However, most girls were moderately active (50% for children and 61.02% for adolescents) or little active (43.75% for children and 27.12% for adolescents). Only 3.76% of tween boys and 1.45% of adolescent boys were reported to be very active (Table 2).

### Physical activity in spare-time score

**Figure 1:**
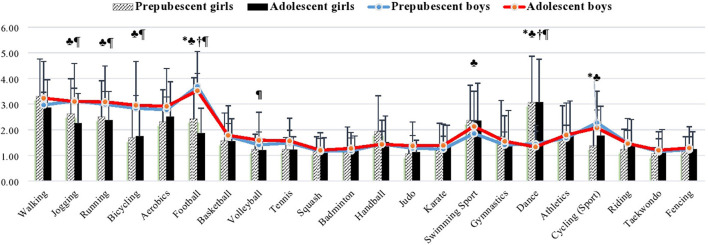
Mean value of physical activity in spare-time for Saudi students stratified by sex and age. ^*^significant difference in prepubescent boys vs. prepubescent girls; ♣ significant difference in prepubescent boys vs. adolescent girls; † significant difference in adolescent boys vs. prepubescent girls; ¶ significant difference in adolescent boys vs. adolescent girls. Physical activity questionnaire for older children PAQ-C (Kowalski et al., 2004), scored on a 5-point scale; 1 corresponds to lowest PA, and 5 corresponds to highest PA. The values of PA are presented as means (±SD).

Students aged 11–14 years in Al-Ahsa governorate engaged in 22 types of physical activities during spare-time, with significantly higher levels among boys than girls (*p* < 0.05; Figure 1). Welch's ANOVA analysis showed that PA scores during spare-time did not differ between groups stratified by sex and age category. Nevertheless, the specific analysis showed that both groups of boys spent more time jogging, running, cycling, and playing football than the group of adolescent girls (*p* < 0.01 in running for both; *p* < 0.001 for the rest). A significant superiority in favor of boys was observed regarding participation in football games for both groups of boys compared with the group of prepubescent girls (*p* < 0.001 and 0.01, respectively), participation in cycling as a sport for the group of prepubescent boys compared to both groups of girls (*p* < 0.05 for all), and participation in playing volleyball for adolescent boys compared to their female counterparts (*p* < 0.01). However, both groups of girls participated in dance significantly more than both groups of boys (*p* < 0.001 for all). Adolescent girls also swam more than boys (*p* < 0.05; Figure 1).

### Physical activity across days of week

**Figure 2:**
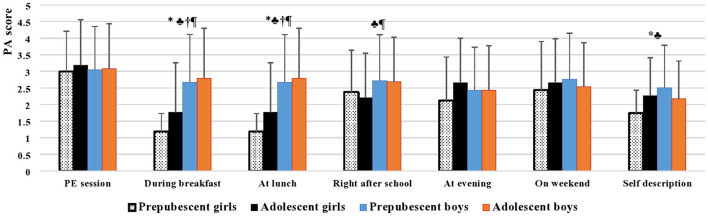
Self-reported physical level and physical activity during physical education class, lunch, right after school, evening and weekend among Saudi students stratified by gender and age category. *significant difference in prepubescent boys vs. prepubescent girls; ♣ significant difference in prepubescent boys vs. adolescent girls; † significant difference in adolescent boys vs. prepubescent girls; ¶ significant difference in adolescent boys vs. adolescent girls. Physical activity questionnaire for older children PAQ-C (Kowalski et al., 2004), scored on a 5-point scale; 1 corresponds to lowest PA, and 5 corresponds to highest PA. The values of PA are presented as means (±SD).

Throughout each day across the seven days of the week, PA scores were significantly higher in boys than in girls (*p* < 0.001) and in both groups of boys compared to the group of adolescent girls (*p* < 0.001 for all; Table 1). Specifically, students' activities during physical education classes, evenings, and the weekend did not differ significantly between all groups stratified by sex and age category. However, both groups of boys were more active than girls during breakfast and lunch periods (*p* < 0.001 for all). A significant difference in favor of boys was also observed between the two groups of boys and the group of adolescent girls regarding after-school PA (*p* < 0.05 for all). Prepubescent boys reported being generally less active during the previous 7 days than their female counterparts and adolescent peers (*p* < 0.05 for all; Figure 2).

**Figure 3:**
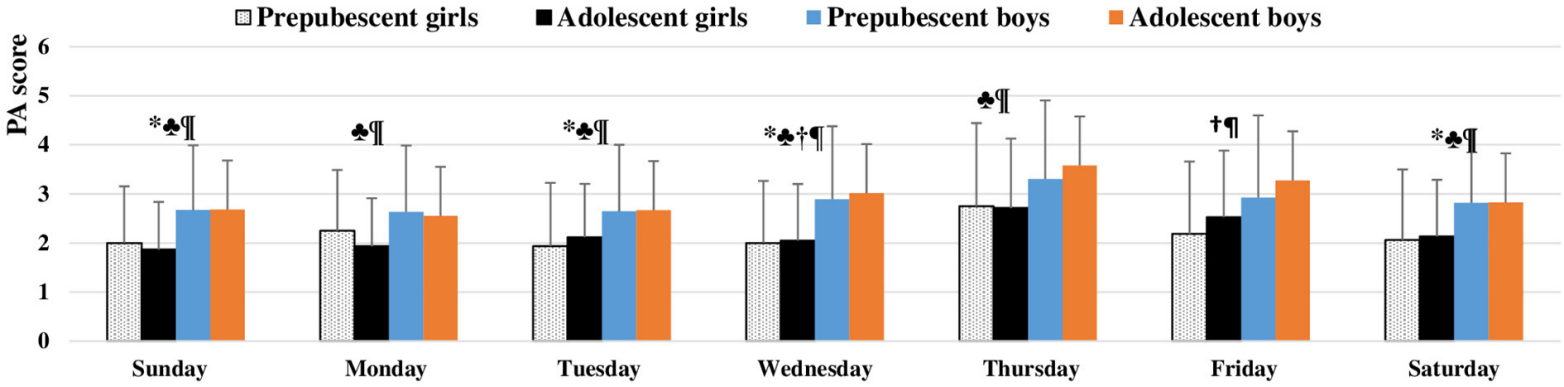
Mean value of physical activity self-report for each day during previous week in Saudi students stratified by sex and age category. * significant difference in prepubescent boys vs. prepubescent girls; ♣ significant difference in prepubescent boys vs. adolescent girls; † significant difference in adolescent boys vs. prepubescent girls; ¶ significant difference in adolescent boys vs. adolescent girls. Physical activity questionnaire for older children PAQ-C (Kowalski et al., 2004), scored on a 5-point scale; 1 corresponds to lowest PA, and 5 corresponds to highest PA. The values of PA are presented as means (±SD).

As shown in Figure 3, adolescent boys were significantly more active than their female counterparts during every day of the week (*p* < 0.05 for Tuesday; *p* < 0.001 for Friday and Wednesday; *p* < 0.01 for the rest); in addition, adolescent boys were more active than prepubescent girls on Wednesdays and Fridays (*p* < 0.05). Prepubescent boys were more active than adolescent girls on all days except Friday (*p* < 0.01 for Tuesday, Thursday, and Saturday; *p* < 0.001 for the rest), and they were more active than their female counterparts on Sunday, Tuesday, Wednesday, and Saturday (*p* < 0.05 for all). There were no statistically significant differences between the two groups of boys or the two groups of girls.

### Sedentary behaviors score

**Figure 4:**
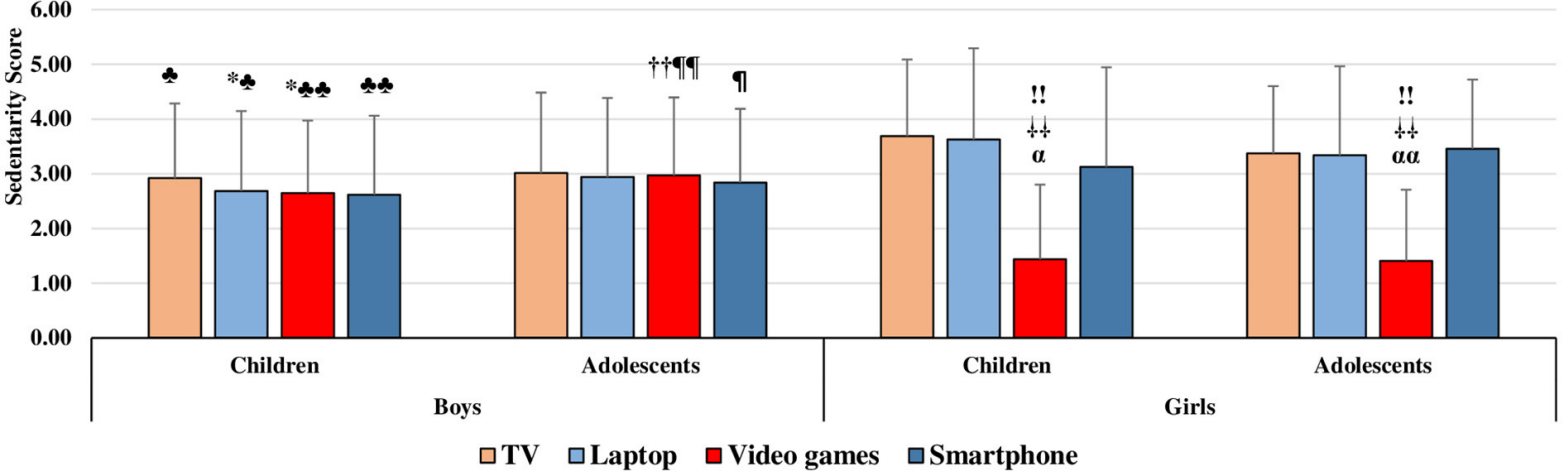
Sedentary behaviors self-report for Saudi students stratified by sex and age category. * significant difference in prepubescent boys vs. prepubescent girls; ♣ significant difference in prepubescent boys vs. adolescent girls; † significant difference in adolescent boys vs. prepubescent girls; ¶ significant difference in adolescent boys vs. adolescent girls. ! differs to laptop use; α differs to smartphone use; 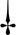 differs to playing video games. Physical activity questionnaire for older children PAQ-C (Kowalski et al., 2004), scored on a 5-point scale; 1 corresponds to lowest PA, and 5 corresponds to highest PA. The values of PA are presented as means (±SD).

The sedentary behavior scores for groups stratified by sex and age category are shown in Table 1. Welch's tests noted similar scores in all groups stratified by gender or gender and age category, respectively. However, significant differences were noted between the groups with respect to the use of various technological devices and the Internet. Compared with both female groups, prepubescent boys watched television and used laptops less (*p* < 0.01 for adolescents regarding laptop use; *p* < 0.05 for others) and played video games more (*p* < 0.01 for children; *p* < 0.001 for adolescents). Adolescent boys also played more video games than both groups of girls (*p* < 0.001) but used smartphones less than their prepubescent counterparts (*p* < 0.01). There were no significant differences between groups of the same gender (Figure 4).

In addition, a significant difference was noted in the prevalence of sedentary behaviors between groups stratified by sex and age category. According to the survey results, 31.92% of boys (10.64% of children and 21.28% of adolescents) and 44% of girls (12% of children and 32% of adolescents) were not sedentary at all or only slightly sedentary. The remaining participants were either sedentary (43.97% of boys and 32% of girls), highly sedentary (20.21% of boys and 22.67% of girls), or very highly sedentary (3.9% of boys and 1.33% of girls; Table 2).

### Predictors of body mass index

Stepwise multiple linear regression analysis showed that a significant regression pattern was identified in the total sample and in the boys, children, and prepubescent boys groups, with R-squared values between 0.012 and 0.055. VIF values were between 1 and 1.152 for all predictors, indicating a lack of collinearity in the results and thus sufficient statistical significance. Table 3 shows the magnitude, direction, and strength of the relationships between individual predictors and BMI in the total sample and in the boys, children, and prepubescent boys groups. PA Across days of week score, as the first predictor that achieved negative beta values, suggests that children aged 11–14 years who were physically active each weekday had a lower risk of obesity. β values were −0.584, −0.847, −0.692, and −0.837, respectively. Sedentary behaviors of the total sample (β = 0.556; *p* < 0.05), mainly video games (β = 0.405; *p* < 0.05), and of the boys' group (β = 0.768; *p* < 0.05), mainly laptop use (β = 0.56; *p* < 0.01), as the second predictor that achieved positive beta values, suggest that students who spent more time in any arousal behavior characterized by energy expenditure ≤ 1.5 metabolic equivalents while in the sitting, lying, or reclining postures had a higher risk of increasing their BMI.

**Table 3:** Stepwise multiple regression analysis models for associations between BMI and physical activity across days of week and sedentary behaviors among Saudi students aged 11–14.

**Model 1**					**Model 2**			
**Variables**		**R^2^**	**β coef. (SE)**	***T***	**Variables**	***R* ^2^**	**β coef. (SE)**	***T***
Total (*N* = 357)	Constant	0.012	18.644 (0.807) ^***^	23.099	Constant	0.03	20.743 (0.838)^***^	24.766
	Sedentary behaviors score		0.556 (0.272) ^*^	2.044	PA across days of week score		−0.584 (0.231)^*^	−0.130
					Playing video games		0.405 (0.195)^*^	0.109
Boys (*N* = 282)	Constant	0.018	18.382 (0.985) ^***^	18.653	Constant	0.055	21.407 (1.069)^***^	20.02
	Sedentary behaviors score		0,768 (0.335) ^*^	2.295	PA across days of week score		−0.847 (0.278)^**^	−3.054
					Use of laptops		0.56 (0.222)^**^	2.52
Children (*N* = 229)					Constant		22.167 (0.889)^***^	24.928
					PA across days of week score		−0.692 (0.293)^*^	−2.360
Prepubescent boys (*N* = 213)					Constant	0.033	22.785 (0.965)^***^	23.611
					PA across days of week score		−0.837 (0.293)^*^	−2.666

Regression β coefficient (unstandardized) represents the degree of change in the BMI for every 1-unit of change in the predictor variable. Model 1 performed with sedentary behavior score; Model 2 performed with sedentary activity levels.

* *p* < 0.05;

** *p* < 0.01;

*** *p* < 0.001. PA, physical activity; SE, standard error.

## Discussion

This study examined the prevalence of physical activity and sedentary behaviors (use of television, laptops, video games, and smartphones) and its effects on BMI in a group of Saudi students aged 11–14 years. The results showed that overweight and obesity were 17.02 and 28.37% in boys, and 9.33 and 0% in girls, respectively. Our results are consistent with those of several other studies in regards to groups of boys (Al-Dossary et al., 2010; Mahfouz et al., 2010; Al-Hussaini et al., 2019), but they diverge dramatically from those studies in regards to groups of girls. Al-Hussaini et al. (2019) reported a respective prevalence of overweight and obesity of 13.4% (14.2% for girls; 12% for boys) and 18.2% (18% for girls; 18.4% for boys) among 7,930 students aged 6–16 years in the city of Riyadh. However, Aliss et al. (2020) found that ~40% of boys and 10% of girls aged 5–15 years in the Jeddah region were overweight or obese.

In addition, our findings noted that boys were more active than girls; however, no significant difference in sedentary behaviors was recorded between boys and girls. The difference in PA scores was consistent, and boys achieved higher levels than girls. The mean value for PA scores for boys was 2.59 ± 0.67; for girls, the mean value for PA scores was 2.29 ± 0.56, which is a similar finding to those of other studies that have used the PAQ-C. Janz et al. (2008) reported a mean PA score of 2.8 ± 0.65 at the age of 13 years old. Crocker et al. (2006) administered a longitudinal study with females and found that the means for PA scores were 2.65 ± 0.59 at the age of 13 years old, 2.42 ± 0.56 at the age of 14 years old, and 2.23 ± 0.69 at the age of 15 years old. Crocker et al. (2003) conducted a longitudinal study with females and found that the means for PA scores were 2.65 ± 0.59 at the age of 15 years old and 2.40 ± 0.55 at the age of 16 years old.

The difference in PA with respect to age has also been reported in several other studies (Nader et al., 2008; Voss et al., 2013; Al-Hazzaa, 2018). However, studies disagree regarding the age at which this difference begins. In the present study, which includes the ages between 11 and 14 years, no significant difference was reported. Telama and Yang (2000), whose sample's ages ranged from 9 to 27 years, reported that the difference started only after the age of 12 years. This study included participants from Finland and used questionnaires as a measurement tool and the difference presented in terms of the frequency of PA and sports participation. Telama and Yang (2000) only reported data of ages at 3-year intervals: 9, 12, 15, 18, 21, 24, and 27.

The present study also attempted to describe PA patterns between boys and girls. In general, the results are consistent with other studies (Gualdi-Russo et al., 2020); girls are less active than boys regarding the mean value of PA. According to the self-report, boys had a mean PA value of 2.59 while girls had a mean PA value of 2.29. The gap in PA level between boys and girls did not differ with age; more specifically, the difference in PA levels for prepubescent boys vs. prepubescent girls did not differ significantly from that of adolescent boys vs. adolescent girls. This contradicted the finding of Nader et al. (2008), who reported on a sample of 1,032 students aged between 9 and 15 years old, that both boys and girls had decreased PA levels as age increased. Thompson et al. (2003) also reported PA levels decreased as age increased for both sexes. Tremblay and Frigon (2005) and McCabe and Ricciardelli (2004) highlight the fact that the timing of pubertal maturation is one of the factors that may contribute to low PA. Belsky et al. (2007) reported that pubertal development for girls at the age of 11 years was associated with a poorer psychological state (depression, global self-worth, perceived athletic competence, maturation fears, and body esteem), which predicted lower enjoyment of PA at the age of 13 and, consequentially, lower moderate-to-vigorous PA levels. It has been suggested that early pubertal maturation may lead adolescents to participate less in PA (Baker et al., 2007).

Interestingly, during PE classes, boys and girls tended to report similar PA effort values between the ages of 11 and 14 years; and only then did small, but statistically not significant, differences between the two sexes begin to appear at the age of 14 years. As the present questionnaire does not provide any information about the intensity of the effort performed by boys and girls during PE sessions, it is difficult to compare our results with other studies, which have found such differences. In a review of 21 studies on PE, boys engaged in moderate-to-vigorous PA for 16–61% of class time, while girls engaged in moderate-to-vigorous PA for 16–57% of class time (Fairclough and Stratton, 2005).

Between boys and girls, there was a significant difference in PA levels during breakfast, at lunchtime, and after school. These differences might result from differences in the availability of equipment and spaces for PA between boys' schools and girls' schools (PE not yet introduced in all-girl schools). During breakfast and lunchtime, tween boys may engage in more unstructured activities than older ones; adolescent boys may engage more in sedentary behaviors, such as reading and talking, instead of engaging in PA. During these times, children choose PA without any restraint and choose their own games (Jarrett, 2002). This may also suggest that boys might show a preference for outdoor activities while girls might prefer indoor activities; this notion is also suggested by Tudor-Locke et al. (2006), who studied sex-specific PA patterns in 81 children (28 boys aged 11 years) using pedometers. Their results indicated that boys showed a preference for outdoor activities as well as more vigorous ones. Another study by Verstraete et al. (2006), which studied 75 boys and 47 girls aged 10 years during unstructured lunchtime periods, reported results confirming the finding of the present study; that is, boys are more active than girls during lunchtime.

Adolescent girls' PA levels decreased after school, suggesting that they were less engaged in structured physical activities. The effect of participation in after-school programs was studied by Gortmaker et al. (2012), who concluded that after-school PA could increase with participation in PA programs. The sample in this present study showed low PA levels during evenings and weekends, and this can be explained by the previous study by Marshall et al. (2004); the children in the present study might have spent their leisure time doing sedentary behaviors. The notion that children spend less time participating in PA while they spend more time performing sedentary activities is known as the *displacement hypothesis* (Sallis, 2000). However, the relationship between physical activity and sedentary behavior is indeterminate (Sallis, 2000).

In addition, our results showed that participant BMI was significantly affected by sedentary behaviors mainly laptop and video game console use. It has been suggested that sedentary behavior is not simply a lack of physical activity, but a set of individual behaviors during which the individual's energy expenditure is similar to that of rest, such as using electronic devices, driving, and reading. It is any arousal behavior characterized by an energy expenditure of ≤ 1.5 METs while sitting or lying down. According to Bartosiewicz et al. (2020), the causes of overweight and obesity in children and adolescents include time allocated to media use, watching movies, or playing games on one's computer, television, or smartphone. Dietz and Gortmaker (1985) were the first to demonstrate the positive relationship between time spent using electronic devices and the prevalence of childhood obesity: the longer a child played on the computer, watched movies online, or used a smartphone, the higher the BMI value. Moreover, Epstein et al. (1995) reported that the incidence of overweight and obesity in children decreased with a decrease in sedentary behaviors, including time spent watching TV or playing computer games. Television screen time was positively correlated with the number of meals eaten and with parental time spent watching programs (Bartosiewicz et al., 2020). Ashdown-Franks et al. (2019), analyzing data from 116,762 adolescents aged 13.8 ± 1.0 years from 41 low- and middle-income countries, found that the prevalence of obesity and sedentary behaviors was lower in low-income countries and higher in upper-middle-income countries. These authors further stated that sedentary behaviors lasting ≥3 h/day were associated with a higher likelihood of obesity in 32 countries and that this relationship was stronger in low-income countries. Two to four h per day of sedentary activity, such as watching television, has also been reported to increase body weight (Nicklas et al., 2001).

Data from the present study support the suggestion that high volumes of PA and low volumes of sedentary behaviors are associated with decreased BMI, which is beneficial to health and requires the identification of appropriate interventions to reduce sedentary behaviors and inactivity (Gualdi-Russo et al., 2020). The relatively large amount of time our participants spent in sedentary behaviors associated with low levels of PA is a possible explanation for the low R-squared values related to the association of these factors with BMI. Gualdi-Russo et al. (2020) reported a decrease in BMI of ~0.007 kg/m^2^ per min per day with a moderate to vigorous increase in PA compared to an increase in BMI of ~0.006 kg/m^2^ per min per day of increased sedentary time. Confirming these results, Katzmarzyk et al. (2015) reported that higher PA values (assessed by accelerometry) were associated with a lower risk of obesity in children aged 9–11 years.

### Study strengths and limitations

The strength of this study lies in its coverage of five different elements of PA. This is different from most studies, which tend to focus on overall PA. The division of the out-of-school element into evening and after-school time is also a unique one. Nevertheless, our findings should be interpreted with an awareness of certain research limits. First, information on eating habits has not been studied. Mustafa et al. (2021) reported that poor dietary habits were the main cause of childhood obesity. Infrequent meals, skipping breakfast, eating in front of the TV screen; drinking sugary drinks, eating without hunger and going out to restaurants were the main reported factors associated with weight gain (Kuzbicka and Rachoń, 2013). Second, although the measurement of PA by self-report is used as a widespread method, it has weaknesses, such as bias and misunderstanding of the questionnaire by some pupils (Armstrong and Welsman, 2006). In this present study, efforts were made to explain the nature of the study to the participants and to encourage them to answer the questionnaire faithfully. Using an objective method for measuring PA is another option, but it is difficult to apply in such a large sample. Third, the questionnaires are unable to describe the intensity and actual duration of PA (Warren et al., 2010). Although there is agreement between researchers on the difference in the value of PA regarding age, there is disagreement on when this difference occurs. For these reasons, more studies are needed to determine when this difference begins, and they should describe the intensity of PA, which will help researchers intervene to raise PA levels, especially for girls. Finally, the number of girls is limited due to the fact that PE has been recently introduced into the education system for girls, and it is not yet generalized in all schools. Completing this study with a greater number of girls could increase accuracy. In addition, the nested data structure should not be overlooked, as it is likely to create dependence in the collected data, leading to negative consequences, such as inflation of standard errors for parameter estimates (Park and Yu, 2016; Miyazaki et al., 2019).

## Conclusions

The results revealed significant differences in the prevalence of overweight and obesity between groups of Saudi students aged 11–14 years stratified by gender and age category. The relative values were 17.02% (19.25% of children and 10.14% of adolescents) and 28.37% (26.76% of children and 33.33% of adolescents) among boys and 9.33% (0% of children and 11.86% of adolescents) and 0% among girls. In addition, the results showed that boys were more physically active than girls. The patterns of PA were changing within elements and between sexes. Boys and girls seemed to differ in the way their PA levels changed throughout the day and week. The PA performed across days of the week was negatively associated with BMI in the total sample and in the boys, children, and prepubescent boys groups. However, sedentary behaviors, primarily video games in all participants and laptop use in the boys' group, had a positive effect on BMI. Therefore, key stakeholders (schools, teachers, professionals, parents) should encourage 11–14-year-olds to be active throughout the week and not just for a few days. They should also ensure that time spent on sedentary activities does not exceed 3 h/d (Ashdown-Franks et al., 2019), primarily video games for both sexes and laptop use for boys. Future research detailing assessments of daily and weekly PA scores in children and adolescents using objective and subjective methods to provide a comprehensive description of PA is of great importance. Future studies involving a large number of girls will also be of great interest and will provide more conclusive results.

## Data availability statement

The original contributions presented in the study are included in the article/supplementary materials, further inquiries can be directed to the corresponding author/s.

## Ethics statement

The studies involving human participants were reviewed and approved by Ethics Committee of the Deanship of Scientific Research, King Faisal University (Ref. No. KFU-REC-2021-OCT-EA00019). Written informed consent to participate in this study was provided by the participants' legal guardian/next of kin.

## Author contributions

MA: conceptualization, methodology, software, validity and reliability of the questionnaire, data analysis, and writing of the original version. He is the guarantor of this work and, as such, has full access to all the data in the study and takes full responsibility for the integrity of the data, and the accuracy of the data analysis.

## Funding

The Deanship of Scientific Research, King Faisal University, Al-Ahsa 31982, Saudi Arabia, financed this study (GRANT623).

## Conflict of interest

The author declares that the research was conducted in the absence of any commercial or financial relationships that could be construed as a potential conflict of interest.

## Publisher's note

All claims expressed in this article are solely those of the authors and do not necessarily represent those of their affiliated organizations, or those of the publisher, the editors and the reviewers. Any product that may be evaluated in this article, or claim that may be made by its manufacturer, is not guaranteed or endorsed by the publisher.
